# Securing the Future of Railway Systems: A Comprehensive Cybersecurity Strategy for Critical On-Board and Track-Side Infrastructure

**DOI:** 10.3390/s24248218

**Published:** 2024-12-23

**Authors:** Nisrine Ibadah, César Benavente-Peces, Marc-Oliver Pahl

**Affiliations:** 1 IMT Atlantique, Campus De Rennes 2 r Châtaigneraie, 35510 Cesson Sévigné, France; 2 DATSI (Department of Architecture and Technology of Informatics Systems), ETS de Ingenieros Informáticos, Universidad Politécnica de Madrid, Campus de Montegancedo, Boadilla del Monte, 28660 Madrid, Spain; cesar.benavente@upm.es

**Keywords:** cybersecurity, railway, cyber physical systems, risk management, critical infrastructure, safety system, vulnerability assessment, digital resilience, threat modeling, certification scheme, review

## Abstract

The growing prevalence of cybersecurity threats is a significant concern for railway systems, which rely on an extensive network of onboard and trackside sensors. These threats have the potential to compromise the safety of railway operations and the integrity of the railway infrastructure itself. This paper aims to examine the current cybersecurity measures in use, identify the key vulnerabilities that they address, and propose solutions for enhancing the security of railway infrastructures. The report evaluates the effectiveness of existing security protocols by reviewing current standards, including IEC62443 and NIST, as well as case histories of recent rail cyberattacks. Significant gaps have been identified, especially where modern and legacy systems need to be integrated. Weaknesses in communication protocols such as MVB, CAN and TCP/IP are identified. To address these challenges, the paper proposes a layered security framework specific to railways that incorporate continuous monitoring, risk-based cybersecurity modeling, AI-assisted threat detection, and stronger authentication methodologies. The aim of these recommendations is to improve the resilience of railway networks and ensure a safer, more secure infrastructure for future operations.

## 1. Introduction

The railway sensors are facing unprecedented challenges in protecting digital actuators and physical infrastructure from cyber threats. With the increasing reliance on digital technology, railways are becoming more vulnerable to a wide range of cyber threats. Cybersecurity of railway sensors includes physical security measures, network security systems, and information security protocols. Rail systems have evolved considerably since their inception, especially in the last decade, which has seen the rise of cybersecurity as a critical component of rail infrastructure and rolling stock. Physical cybersecurity must be handled with extreme vigilance [1]. Increasing digitalization makes the system more intelligent and allows consumers to better benefit from innovative services. At the same time, digitization also makes sensors more critical that creates significant risks [2], as increased exposure to cyber-attacks and cybersecurity incidents potentially puts the security and privacy of sensors data at serious vulnerabilities. Thus, it is important to increase cybersecurity in railways and its impact on the physical actuators by:

- Preventive Techniques: Implement multiple cybersecurity measures to prevent attacks;
- Risk Assessment: Know the potential risks and assess them regularly;
- Cybersecurity Culture: Develop a culture of cybersecurity awareness throughout the organization.

Digital innovation has already delivered major breakthroughs in efficiency, comfort, and safety across the rail sensors [1]. As technologies modernize, their exposure to cyber risks increases [3]. The rail industry prioritize cybersecurity to effectively manage security and safety threats of trackside sensors, to on-board actuator networks. As hackers use malware, viruses, and social engineering to attack rail systems, operators innovate to stay ahead of these challenges. While rail assets are designed to last for decades, cyber threats are constantly evolving before a project even begins, manufacturers must plan to rethink a product’s architecture approximately every five years to ensure adequate protection throughout its 30-year lifespan. If anticipation is the key to effective cybersecurity, companies must know how and what to anticipate to make the rail sensors more resilient to cyberattacks. Manufacturers must work with other transportation operators and external experts to create an open environment for discussing common threats. Railway sensors are complex and interconnected, requiring a robust security approach to protect critical actuators and prevent potential threats such as terrorism, cyber-attacks, and natural disasters. The implementation of the Secure-by-Design approach in railway systems involves several steps, including:

- Identify threats, vulnerabilities, and risks for each on-board sensor;
- Design security controls and architecture of each actuator;
- Implement and test security controls;
- Maintain and update the security system.

This paper is in line with some research topics related to cybersecurity and railway, with specific objectives to answer some research questions, like:

- How can we define an appropriate classification of cybersecurity issues and problems of rail physical infrastructure?
- What are the adopted mechanisms for current risk management?

Current approaches are often insufficient to address the specific challenges of integrating modern and legacy rail systems, particularly through the development of standards such as IEC 62443 [4] and the NIST Cybersecurity Framework. Current studies and protocols typically focus on isolated railway infrastructure components and neglect a holistic approach that combines cyber and operational security. In addition, especially in environments where legacy systems are interconnected with new technologies, many frameworks lack the adaptability to address emerging threats from advanced persistent threats (APTs), creating security blind spots. A major limitation remains the lack of a comprehensive, real-time cybersecurity framework tailored to the specific needs of railway systems.

Research Gap: Although considerable advances have been made in confronting the cybersecurity issues associated with railways, significant shortcomings persist in the effective integration of both contemporary and legacy systems. The current approach, as exemplified by the standards set forth in IEC 62443 and the NIST Cybersecurity Framework, frequently focuses on discrete infrastructure elements without integrating a unified strategy that encompasses both cyber and operational security. Furthermore, a considerable number of frameworks are deficient in their capacity to adapt effectively to emerging threats, such as advanced persistent threats (APTs), particularly in environments where legacy systems are present alongside novel technologies. There is a pressing necessity for the creation of a comprehensive, real-time cybersecurity framework that is specifically tailored to address the unique cybersecurity challenges faced by the railway industry, ensuring comprehensive protection through seamless integration and continuous monitoring of evolving threats. This paper aims to fill this gap by proposing a multi-layered security framework that addresses both current and legacy system vulnerabilities. This paper aims to fill this gap by proposing a multi-layered security framework that addresses both current and legacy system vulnerabilities. The study not only identifies critical gaps, but also provides new risk management solutions by reviewing recent advances in cybersecurity standards and conducting a detailed analysis of cyberattacks on railway infrastructure. These solutions provide a more resilient approach to securing rail systems against evolving cyber threats, with a focus on continuous monitoring, AI-driven threat detection, and stronger integration of cybersecurity with operational safety protocols.

The detailed answers to all these questions are highlighted in the present document, which is organized as follows. Section 2 presents understanding systems, threats, and infrastructure of railway cybersecurity. Section 3 highlights comprehensive cyber strategies for railway systems. Section 4 presents risk management strategies for railway infrastructure to mitigate cybersecurity threats. In Section 5, we discuss emerging threats and future security trends. Finally, we finish with the conclusion and some future works.

## 2. Understanding Railway Cybersecurity: Systems, Threats, and Infrastructure

**Figure 1:**
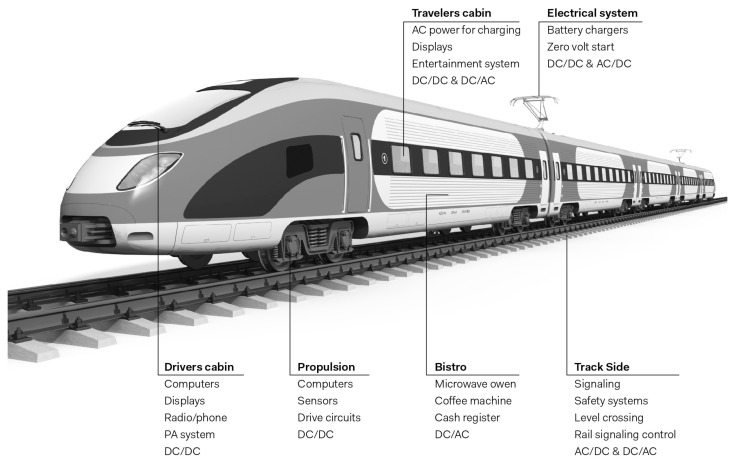
Example of On-board/Trackside instances.

Railway systems are mainly composed of multiple types of sensors. The control of these elements makes the engine more fluid and remotely manageable to monitor the real and the actual situation of each on-board sensor, as shown in Figure 1. This field is more complex in the presence of diverse sensors with specific functionalities. All on-board instances must be highly controlled in real time to prevent all dramatic incidence that can touch live passengers.

The railway trackside is composed of multiple electronic devices interconnected to ensure safety and improve operational efficiency. Meanwhile, the trackside sensors are directly controlling the onboard sensors. Some of the existing railway instances are presented below.

### 2.1. On-Board Sensors

Trains rely on a variety of sensors to collect data about the train’s performance, the track, and the environment. Some common sensors installed on trains include:

1. Speed sensors measure the train’s velocity and provide critical data for controlling and ensuring safe operation;
2. Position sensors track the train’s location, ensuring correct routing and monitoring movement;
3. Temperature sensors monitor both the interior and exterior conditions of the train, optimizing passenger comfort and safeguarding the equipment against extreme temperatures;
4. Pressure sensors measure the pressure within the train’s brake systems and wheels to ensure proper functionality, while also detecting any potential issues with the wheels or axles;
5. Accelerometers measure the train’s acceleration and deceleration to identify issues such as wheel slip or brake fade;
6. Tilt sensors measure the train’s tilt and detect any stability issues that could lead to derailment;
7. Obstacle detection sensors detect objects on the track, like debris or fallen trees, ensuring passengers’ safety;
8. Obstacle avoidance sensors used in autonomous trains to detect and avoid obstacles, which also contributes to passenger safety;
9. Infrared sensors specifically designed to detect the presence of individuals, animals, or other vehicles on the tracks ahead of the train.

All of these sensors are linked to a central control system that analyzes the data and determines how the train should be operated. This permits the train’s operator to modify the train’s speed, braking, and other functions in real time to guarantee the safety of both the train and its passengers.

### 2.2. Trackside Sensors

Railroad trackside sensors gather data about the track condition, surrounding environment, and trains. Common sensors used to trackside include:

- Track geometry sensors measure the shape and alignment of railway tracks to detect issues such as misalignment or irregularities;
- Trackbed sensors measure trackbed conditions, including soil moisture, temperature, and vibration;
- Weather sensors measure temperature, humidity, rainfall, wind speed, and direction;
- Track switch sensors are designed to detect the position of switches to ensure that trains are directed to the correct track;
- Train presence sensors are used to detect the presence of trains on the track, to promote safe railway operation and prevent collisions;
- Train speed sensors measure the velocity of trains in the trackside area to ensure safe operation and identify performance issues;
- The obstacle detection sensors identify fallen trees or debris on the track;
- Fault detection sensors detect any issues with the rail track or surrounding environment, such as broken rails or landslides.

### 2.3. Sensors Categories and Interactions

Grouping the sensors into categories based on their functions would enhance clarity and readability. Potential categories include:

- Safety Monitoring Sensors are designed to detect potential safety issues that could impact railway operations. They include:
  - Track Condition Sensors: Monitor track deformation, wear, and breaks.
  - Obstacle Detection Sensors: Identify objects on the tracks using radar, LiDAR, or optical cameras.
- Environmental sensors are designed to measure external conditions that could potentially impact the railway system. They include:
  - Weather sensors: in place to track temperature, humidity, and precipitation, which could impact track integrity or train performance.
  - Vibration sensors: used to monitor external vibrations that could affect infrastructure stability.
- Train Operational sensors: These measure the performance and state of the train, namely:
  - Speed Sensors: real-time monitoring of train speed for braking systems and signaling coordination.
  - Brake Sensors: measure brake pressure and performance to ensure optimum operation.

These sensors are interconnected with each others to interact within the broader rail system. This could include:

- Sensor networks: both on-board systems and trackside infrastructure operate together to provide a holistic view of rail operations. As an example, speed sensors and brake sensors work together to ensure safe braking, while trackside sensors coordinate with on-board systems to provide real-time data on track conditions and signal status.
- Centralized control systems: these sensors feed data to a central control system, where decisions about train routing, speed adjustments, or emergency stops are determined. The interaction between on-board and trackside sensors is critical to the safety and efficiency of the system.

**Figure 2:**
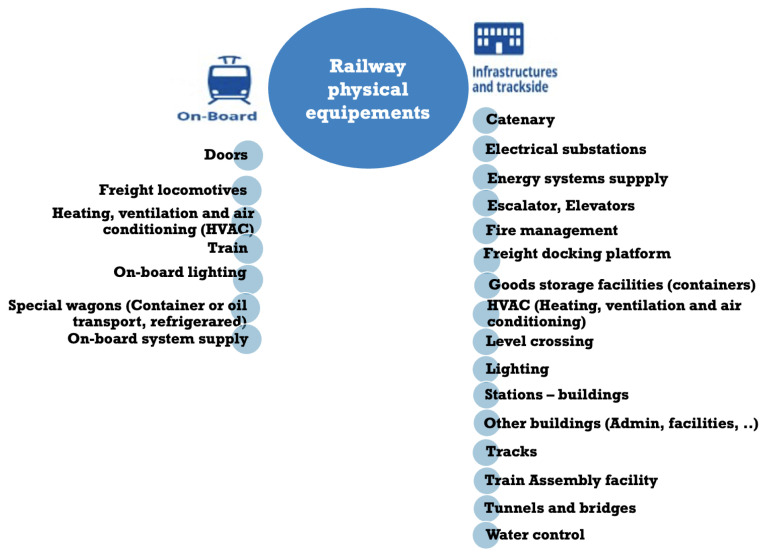
An overview of the railway physical infrastructure.

Figure 2 simplifies the railway system view of all these sensors that are interconnected with a set of servers that evaluates the data and makes decisions. This complex infrastructure makes the whole system more vulnerable to functional attacks and safety management.

The including insights on emerging sensor technologies that mainly impact railroad cybersecurity, as for:

- LiDAR and optical sensors provide high-accuracy environmental and obstacle detection, thereby improving safety. However, their reliance on data-intensive communications makes them vulnerable to cyberattacks that could manipulate or corrupt the transmitted data.
- Smart sensors employ AI and machine learning to predict potential failures (e.g., track deformation) and adjust in real time. However, their complexity exposes new attack surfaces for cybercriminals to exploit.
- Wireless networks minimize the need for physical connections, but can introduce vulnerabilities in data transmission, especially if encryption and authentication protocols are not sufficiently robust.

The implementation of LiDAR, smart sensors, and wireless sensor networks in railway systems offers enhanced capabilities for real-time monitoring and predictive maintenance. However, these emerging technologies also contribute to an increase in system complexity and a potential expansion of the attack surface for cybercriminals. This, in turn, necessitates the integration of more robust cybersecurity measures, including end-to-end encryption and real-time anomaly detection. By organizing the sensor categories in a systematic framework and elucidating their interrelationships, as well as by examining emerging technologies and the cybersecurity challenges they may pose, this section aims to elucidate the implications of sensor vulnerabilities in the broader railway infrastructure.

## 3. From Safety to Security: Comprehensive Cyber Strategies for Railway Systems

### 3.1. Rail Cybersecurity vs. Rail Safety

For railway architecture, safety and cybersecurity share the same goal: to ensure that passengers and employees are not put at risk and that trains run on schedule. However, the two concepts should not be confused [5], or they will not be adequately addressed. In the rail industry, safety is generally defined as protecting passengers and systems from unintentional harm, such as deficient mechanisms, faulty lights, system bugs, and so on. On the other hand, cybersecurity is not about safety but about security: it is about protecting people and infrastructure from intentional harm caused by people with malicious intent. Unlike physical security, which is based on proven principles, cybersecurity never rests on its laurels. It must always stay one step ahead of increasingly sophisticated attackers. To ensure IT security, the rail industry must continually improve and enhance its cybersecurity solutions to meet the next emerging threat. A major obstacle to effective rail cybersecurity, however, is the difficulty of reconciling proven security protocols with cybersecurity systems that must constantly evolve.

Ref. [6] offers an exhaustive examination of the security issues associated with CPS, with particular emphasis on threat models, attack methodologies, and strategies that can be deployed to enhance the security of railway systems. It draws attention to the fact that attacks on cyber–physical components have the potential to compromise the overall safety and reliability of infrastructure. In considering the impact of cyberattacks on railway safety and cybersecurity, it is essential to examine the concept of Cyber–Physical Systems (CPS) and their inherent security challenges, as presented in this paper. Given the complex nature of railway infrastructures, which comprise both physical components (e.g., on-board sensors and trackside equipment) and digital systems (e.g., communication networks and control systems), the security of these cyber–physical systems is of paramount importance. The occurrence of cyberattacks on railway systems can have direct and severe impacts on both safety and operations. This is due to the fact that such attacks may lead to the compromise of the physical functioning of critical components. Table 1 provides a non-exhaustive collection of significant disclosed cybersecurity incidents that impacted transportation operations or threatened or had the potential to threaten transportation security. Note that we do not include cyber incidents that resulted solely in the theft or compromise of data. While the earliest incidents may have directly impacted transportation operations and safety, the most recent ransomware attacks did not affect critical rail sensors, but they did significantly disrupt transportation services.

**Figure 3:**
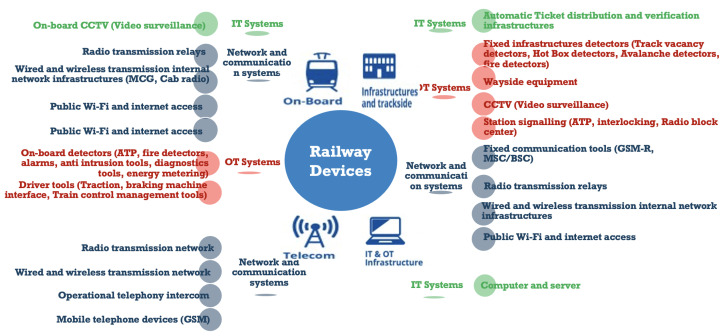
Railway sensors.

A closer examination of the incidents reveals that the sensors most affected are ticketing, passenger information, CCTV, on-board Wi-Fi, and entertainment systems [25]. Fortunately, in the vast majority of cases, operational technology (OT) systems are secure, and the problems were either minor or unknown. These examples show that rail IT systems have been the primary focus. However, as systems become more interconnected, signaling, control, and telematics are increasingly vulnerable to such attacks, especially with the diversity of rail sensors, as shown in Figure 3.

To provide a comprehensive analysis of the case studies presented in the article, it is essential to delve deeper into the specific threats, vulnerabilities, and technical intricacies associated with each cybersecurity incident. The following points illustrate a more detailed technical examination of these incidents and the underlying technologies involved.

1. Deutsche Bahn Ransomware Attack (May 2017)
  - Threats and Vulnerabilities: The exploitation of the EternalBlue vulnerability in legacy Windows systems was facilitated by inadequate patch management and insufficient network segmentation.
  - Underlying Technology: The EternalBlue exploit is a vulnerability in the SMB (Server Message Block) protocol. This allows ransomware to spread across unpatched systems in a lateral movement.
  - Strategies Used: The failure to effectively manage patches allowed the proliferation of ransomware. The lack of sufficient network segmentation permitted the attack to affect the operation of multiple systems.
  - Technical Takeaway: The necessity of prompt patch management and effective network segmentation to impede the propagation of ransomware across critical systems is paramount.
  - Linking to Broader Railway Security: The analysis underscored the susceptibility of non-critical systems and the imperative for prompt system updates and comprehensive cybersecurity strategies.
2. Swedish Transport Administration DDoS (October 2017)
  - Threats and Vulnerabilities: The absence of a robust DDoS mitigation infrastructure is a significant vulnerability. Centralized systems that lack sufficient load balancing and traffic filtering are particularly susceptible to DDoS attacks.
  - Underlying Technology:
    - Botnets: Attackers employed the use of a botnet to generate a substantial amount of traffic, overwhelming the system.
    - Centralized systems: These systems are susceptible to overloading due to the absence of adequate DDoS protection measures.
  - Strategies Used: The objective was to flood the network with malicious traffic in order to exhaust server resources. The mitigation strategy involved collaboration with the Internet Service Provider (ISP) for traffic filtering.
  - Technical Takeaway: It is imperative to implement a robust cybersecurity strategy that encompasses DDoS protection, load balancing, and traffic filtering to guarantee the resilience and uninterrupted operation of critical railway IT systems.
  - Linking to Broader Railway Security: It was demonstrated that there is a necessity for the implementation of DDoS protection and the establishment of alternative communication channels within the railway industry. Furthermore, the significance of ensuring the resilience of IT infrastructure was emphasized.
3. Ukrainian Railway Cyberattacks (February 2022)
  - Threats and Vulnerabilities: The state-sponsored APTs were targeted at control systems, communication, and ticketing. Furthermore, communication protocols were found to be vulnerable to exploitation.
  - Underlying Technology: APT malware and phishing attacks have been observed to exploit weaknesses in man-in-the-middle attack defenses for communication protocols.
  - Strategies Used:
    - Multi-vector attacks were directed at both operational and customer systems.
    - International agency collaboration for cyber defense.
  - Technical Takeaway: It is imperative to implement geopolitical risk management strategies, utilize encrypted communication channels, and foster international collaboration in order to enhance railway cybersecurity.
  - Linking to Broader Railway Security: It was demonstrated that critical infrastructure is vulnerable during conflicts and that there is a need for enhanced cyber resilience, international cooperation, and redundant systems.
4. Trenitalia Ransomware Attack (March 2022)
  - Threats and Vulnerabilities: The attack was of the phishing variety and delivered ransomware. The network lacked segmentation and endpoint security.
  - Underlying Technology:
    - Ransomware encryption: The encryption of critical databases, rendering them inaccessible.
    - Phishing attack: A social engineering tactic designed to gain unauthorized access to internal systems.
  - Strategies Used: The phishing attack was directed at employees, and the inadequate segmentation of systems permitted the ransomware to propagate beyond the customer environment.
  - Technical Takeaway: The necessity of phishing awareness, endpoint security, and segmentation in order to prevent the adverse effects of ransomware on critical infrastructure must be enhanced.
  - Linking to Broader Railway Security: It was emphasized that network segmentation, robust backup systems, and comprehensive incident response plans are essential for the minimization of disruption.
5. Danish Rail Cyberattack (October 2022)
  - Threats and Vulnerabilities:
    - The company’s reliance on third-party vendors for real-time data are a significant vulnerability.
    - Furthermore, the absence of robust contingency and backup systems for operational data represents a substantial risk.
  - Underlying Technology:
    - Vendor exploit: Attackers gained unauthorized access to real-time train location data.
    - Data availability issues: The absence of a backup system for train location data.
  - Strategies Used: The exploitation of the vendor was directed towards external systems. In order to mitigate the effects of this exploitation, a decision was made to switch to manual controls in order to maintain operational continuity.
  - Technical Takeaway: The necessity of third-party risk management, security audits, and backup data systems to guarantee the continuity of operations during cyber incidents is of paramount importance.
  - Linking to Broader Railway Security: It demonstrated the vital role of third-party risk management and the necessity for backup systems to guarantee operational sustainability in the event of a cyber incident.

By examining these case studies, the article provides a more profound understanding of the particular technologies, vulnerabilities, and attack strategies that were successfully exploited in each incident. This analysis facilitates comprehension of the methods employed by attackers to circumvent network defenses and to elucidate the technical solutions (e.g., patch management, DDoS protection, endpoint security, vendor management) that can be implemented to safeguard critical railway infrastructure. These incidents demonstrate the growing susceptibility of railway infrastructures to cyberattacks, encompassing a range of threats, from ransomware to sophisticated attempts to disrupt signaling systems. By way of illustration, Table 2 provide valuable insight into of the latest case studies of the tangible effects of cyberattacks in the real world. These studies offer a comprehensive comparison of the financial impact of such incidents and emphasize the cost-effectiveness of mitigation strategies.

They underscore the necessity for robust cybersecurity measures, proactive risk assessments, and the implementation of secure communication protocols to safeguard critical rail systems from evolving threats. Integration of these details transforms this article from an overview into an in-depth discussion on cybersecurity strategies for railways, thereby enhancing its robustness and applicability to current network security challenges.

### 3.2. Cybersecurity Issues and Problems Classification

An interdependent group of stakeholders operates, manages, and maintains the rail infrastructure. A secure and safe environment for sharing data and computational models, and for storing, accessing, and processing data over the long life of the assets, will facilitate the creation of appropriate decision-support tools. The first steps in creating such an environment will be an analysis of the needs of the railway stakeholders, interdependencies, processes, maintenance layout, etc. The classification of problems and difficulties formed during this study has been categorized into three categories: governance, business, and technology, as shown in Table 3.

There are numerous cyberattacks that can corrupt or damage physical or digital infrastructure, causing everything from minor disruptions to loss of life in terms of operations and equipment. Cybersecurity is a continuous effort; the security team must close all potential gaps, while attackers only need to identify a few openings. Adequate security coverage depends on updating both technological and human factor requirements and requires addressing numerous system design areas. It must also be continuously monitored and improved. To achieve confidentiality, integrity, and availability, and to balance the security objectives of the collaborators for access control, information transmission, and data storage, multiple layers of security are needed to provide overall security for the collaboration and sharing of information sharing among the railway partners. A unified information security system that outlines security protocols and requirements should integrate all security requirements. The operation of railway systems depends on the use of a variety of communication protocols that facilitate the exchange of data between different components, including sensors, actuators and control systems. Among these, the following protocols are particularly prevalent:

- MVB (Multifunctional Vehicle Bus) is a communication network deployed in trains to connect subsystems such as those controlling the braking, signaling, and other safety-critical functions. However, despite its safety-critical applications, the MVB is vulnerable to interception and spoofing attacks.
- CAN (Controller Area Network) is a communication protocol frequently utilized in train control systems. It enables interconnection between various embedded systems. However, CAN is vulnerable to message replay and arbitration attacks, wherein a malicious actor gains control of the communication bus and modifies the transmitted data.
- TCP/IP is employed in the context of broader railway communication networks, including those between central control systems and maintenance stations. Potential vulnerabilities associated with TCP/IP include Denial of Service (DoS) attacks and unauthorized access, which may occur if the necessary security measures are not implemented effectively.

Even for hardware, we must take the following into consideration:

- Vulnerability of Physical Devices: It is common for railway systems to utilize a range of hardware components, including sensors, actuators, and communication devices. The potential for these tangible items to become susceptible to security breaches is heightened when their physical composition is not adequately protected against the evolving nature of modern cyber threats.
- Secure Hardware Design: Research into secure hardware design is ongoing, with a particular focus on the creation of tamper-resistant devices and the integration of security features directly into the hardware. One example of such a security feature is the Trusted Platform Module (TPM).

By elucidating the prospective ramifications of digital attacks on the operations and safety of railway systems, it is possible to a gain greater comprehension of the gravity of the situation. Such considerations may include:

- Operational disruptions: A cyberattack has the potential to result in system outages, which could in turn lead to train delays or cancellations. To illustrate, ransomware attacks have the potential to render operators unable to access critical systems, resulting in considerable disruption to the provision of services.
- Safety Risks: It is important to consider how cyber-related incidents could potentially compromise safety systems, for instance, train signaling or braking systems, which may ultimately result in accidents. One such example would be an attack on signaling systems, which could lead to train collisions or derailments.
- Financial Impact: The financial impact of cyber incidents must be emphasized. This encompasses the costs associated with recovery, legal liability and reputational damage. It has been demonstrated that a significant cyber incident can result in a financial loss of millions of dollars for railway companies. This is due to the combined effect of direct and indirect costs incurred. A predictive analytics for future threats by forecasting vulnerabilities and financial impacts helps in proactive planning, as presented in Table 4.

### 3.3. Technical Strategies to Mitigate Network Security Measures

In order to demonstrate the implementation or enhancement of network security measures for railway sensors and actuators, we propose an extension of the existing section. This expansion would include the following technical aspects:

1. *Network Security Architecture for Railway Systems*: This solution may be applied used several technics, like:
  - The *Segmentation of Networks* is of paramount importance to safeguard against potential breaches. In railway systems, operational technology (OT) and information technology (IT) networks often coexist, necessitating a robust approach to network segmentation. This strategy aims to impede the lateral movement of attackers between different systems. The implementation of virtual local area networks (VLANs), for instance, can effectively segregate sensitive OT components—such as train control systems or trackside sensors—from less critical IT systems, thus reducing the potential for compromise.
  - The implementation of more secure protocols such as *MQTT* (*Message Queuing Telemetry Transport*) *with encryption* (*e.g., TLS*) can assist in securing communications within railway systems.
  - It is imperative that *firewalls and intrusion detection systems* (*IDS*) are deployed at pivotal points within the network. These systems are tasked with monitoring and filtering both incoming and outgoing traffic, with the objective of identifying potential cyberattacks. Such attacks may include denial-of-service (DoS) attacks or attempts to access the network without authorization. To illustrate, one may deploy tools such as Snort or Suricata, which have the capability to analyze network traffic in real time and generate alerts when anomalies are detected.
2. *Technical security measures for sensors and actuators*: Specific techniques may be employed to enhance the security of sensors and actuators in railway systems. These include:
  - The use of *encryption*, which can be implemented end-to-end for data transmitted between sensors, actuators, and control systems. The purpose of this is to prevent eavesdropping and data manipulation, as exemplified by the use of AES-256 encryption for communication between onboard sensors and central control units. This ensures that even if a transmission is intercepted, the data remain protected.
  - The *authentication* in order to ensure the security of the network, it is necessary to implement multifactor authentication (MFA) for all devices and users. The integration of MFA with behavioral biometrics and passwordless authentication can mitigate vulnerabilities while concurrently reinforcing security. This is especially pertinent in sectors such as transport and infrastructure, given the potential consequences of data breaches in such fields.
  - The *Access Control* like Role-Based Access Control (RBAC) must be employed to guarantee that only authorized personnel can access critical systems. The use of X.509 digital certificates for device authentication is also recommended in order to prevent unauthorized sensors and devices from communicating with control systems.
3. *The improvement of security protocols*: It is recommended that improvements are made to existing railway network protocols. Such improvements could include, for example:
  - It is essential to implement a *regular firmware update* system for sensors and actuators. This system should be designed to facilitate remote firmware updates, ensuring that all devices are running the latest security patches. Furthermore, a secure boot mechanism should be employed to verify the integrity of the firmware during the boot-up process. This mechanism will prevent the inadvertent application of unsanctioned updates.
  - *Vulnerability scanning and penetration testing* are essential components of any robust cybersecurity strategy. These processes should be performed on a regular basis to identify potential weaknesses within the network and address them proactively. The utilization of sophisticated tools, such as Nmap for network scanning and Metasploit for penetration testing, can facilitate the simulation of cyberattacks and an evaluation of railway networks’ resilience against potential threats.

The implementation of these techniques in a railway context underscores the necessity for a multi-layered security strategy that encompasses the integration of contemporary technologies and industry best practices, with the objective of protecting railway sensors and actuators. By employing encryption, authentication, firewalls, and AI-driven monitoring, railway networks can fortify their resilience against an array of cyber threats. This approach offers greater technical depth and actionable insights, enabling the rail industry to discern the specific steps necessary to bolster security measures in railway systems. However, it is essential to consider the potential challenges and limitations that may emerge during the implementation of any proposed strategy. In this regard, the following points of Table 5 merit particular attention.

It is also imperative to guarantee that each proposed security measure includes continuous monitoring and auditing. To illustrate, following the implementation of network segmentation, it is recommended that quarterly audits be conducted to confirm the absence of unauthorized devices or users accessing sensitive network segments. The utilization of automated tools, such as SIEM (Security Information and Event Management), is an effective approach for the real-time tracking and analysis of security logs. For more details of the previous table, Table 6 is a detailed table showing return on investment (ROI) estimates for cybersecurity measures that address specific challenges presented previously in Table 5. The table includes both the cost of implementation and the estimated savings from mitigating cyber threats.

The comprehensive guide to railway operators presented in our paper is based on a detailed analysis of the proposed strategies, including their associated challenges, limitations, and mitigation strategies. Our approach ensures that the recommendations provided are actionable and practical, offering professionals a clear understanding of how to enhance their network security in real-world operations.

Section 4 provides a more comprehensive view of the challenges and practices in safeguarding railway infrastructure against cyber threats, through the incorporation of these enhancements. This approach serves to highlight the significance of this research to engage practical insights and implications to ensure a resilient cyber–physical system security.

## 4. Mitigating Cybersecurity Threats: Risk Management Strategies for Railway Infrastructure

### 4.1. Security Risks, Threats, and Vulnerabilities

Recent serious security events involving critical infrastructure, including railways, demonstrate that the risks are real and that immediate action is needed to address them. In response, governments have enacted legislation requiring operators of critical systems to implement “state-of-the-art” security practices. While rules and regulations are in place, there are still no industry-wide practices or guidelines on how to implement these measures in the rail environment. Facing the security challenges of the rail industry is necessary for:

- *Infrastructure operators:* Continuous, safe traffic operations depend on the ability to quickly detect and resolve threats to telecommunications, train control, and signaling systems. Complete network visibility and the ability to identify and remediate threats as they occur help minimize operational uncertainty and disruption.
- *Rail operators:* Rail operations depend on the authenticity of the many signals and commands sent over today’s vulnerable communications channels. Continuous monitoring, immediate notification of deviations from standard conditions, and recommendations for incident resolution enable effective risk mitigation and business continuity.
- *Original equipment manufacturers:* The modernization of critical systems introduces significant risks to systems that were originally designed to operate for decades. Incorporating cybersecurity into the design of modern systems by security-by-design or the upgrade of legacy systems minimizes these risks, anticipates compliance with railway standards, and improves the level of integrity and security.

There is a lack of practical advice on how to incrementally integrate cybersecurity into both old and new systems. Service providers need to develop real-world cybersecurity expertise. Over time, industry and academia will need to share this with partners for international rail operators. We need to have answer to the questions “How do you start?” and “How much money and resources are there?” The parameters of the security risk formula are as follows:

SecurityRisk=(Likelihood)×Impact=(Threats×Vulnerabilities)×Impact

In the context of railway network security, a notable shortcoming is the lack of comprehensive guidance on how to quantify risks and establish risk priorities. The following elements are to be included:

1. *Quantifying Risks*: The quantification of risks in the field of cybersecurity entails the attribution of numerical values to potential threats and vulnerabilities, with a view to gauging their overall impact and likelihood of occurrence. This is achieved through the application of risk scoring methodologies, exemplified by the Common Vulnerability Score System (CVSS) and risk matrices. To elucidate this process, risk scoring and the risk matrix are necessary.
2. *Determining Risk Priorities*: In order to ascertain the relative risk priorities, it is possible to use a risk assessment framework that takes into account both the potential severity of impact and the probability of occurrence. For a more detailed account of this process, please refer to the following steps:
  Step 1: Identify assets and threats;
  Step 2: Estimate the likelihood and impact;
  Step 3: Calculate risk score;
  Step 4: Prioritize risks.
3. *A table example of quantifying and prioritizing risks*: Table 7 provides an illustrative example of a method for quantifying and prioritizing risks to render the process more actionable. The application of risk quantification has highlighted several potential challenges associated with the implementation of these methodologies. These include, for instance:
  - *Incomplete Data*: The collection of sufficient data to evaluate the probability of specific threats may prove challenging, particularly in the case of emerging threats such as advanced persistent threats (APTs).
  - *Subjectivity*: The process of estimating the potential impact and likelihood of a phenomenon is not infallible and may be influenced by subjective factors. The data employed and the experience of the assessor can both play a role in determining the subjectivity of the estimation.
  In [26], the team of Rubia Fatima and colleagues have explored *game-based education* as a potential solution for combating these types of attacks. The study demonstrates the potential of game-based education as an innovative approach to counteract social engineering and phishing attacks. By immersing users in simulated situations, game-based learning can facilitate their ability to identify threats in a structured setting, thus enhancing the learning experience. To illustrate, the incorporation of simulation games that emulate social engineering attacks can facilitate users’ capacity to identify and respond to such threats in an efficacious manner. This approach not only renders training more engaging, but also enhances the retention of information.
4. *Solution*: In order to address this issue, it is recommended that frameworks such as *the NIST Cybersecurity Framework* and *ISO/IEC 27005* be employed for the purpose of prioritization. The NIST framework offers detailed guidance on the identification and prioritization of risks through the assessment of asset criticality and the threat landscape. Furthermore, ISO/IEC 27005 provides a comprehensive set of steps for conducting risk assessments specifically for information security management systems (ISMS), offering a structured approach to the quantification and prioritization of risks. A detailed discussion of these norms is highlighted in the next subsection.

### 4.2. Risk Management in the Railroad Industry

Industry regulators are increasingly looking to protect railway systems from cyber threats. Regulations vary in different countries, but are generally designed to mitigate the risk of cyberattacks and improve security across railway systems. Discussions were held with relevant railway stakeholders to identify the most popular risk management techniques currently used by RUs and IMs. During these sessions, the participants made their choice of methods. As required by standards that include human factors, an independent cybersecurity assessment can be performed on any equipment or part of the rail system that sends, processes, or receives data to ensure that these data have not been altered, corrupted, or intercepted for inappropriate use. Table 8 shows good frameworks from the cybersecurity literature.

**Figure 4:**
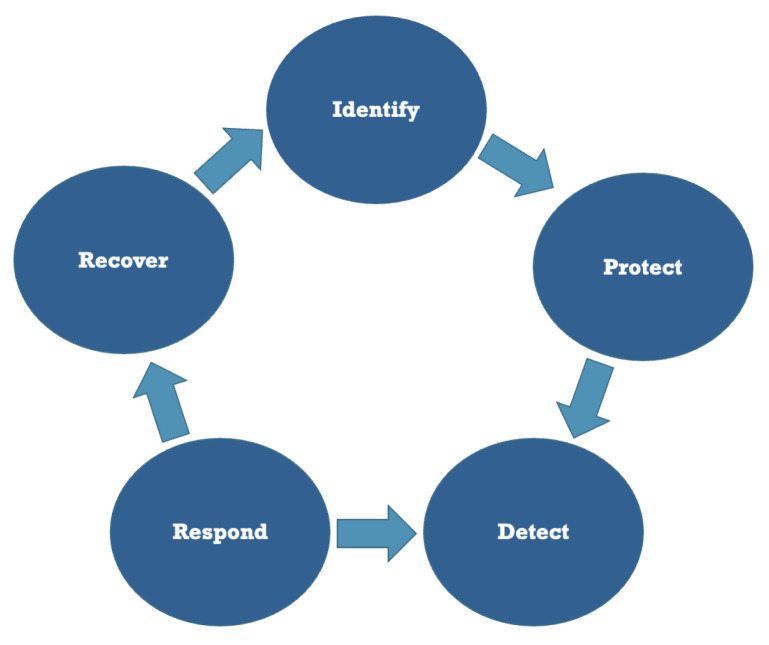
NIST CSF phases.

IEC 62443 and prTS 50701 are two examples of great security recommendations, standards, and frameworks. The NIST Cybersecurity Framework (NIST CSF) [5] is used in this study to discuss how to address the difficulties. A brief overview of the business drivers is followed by a discussion of the security measures that need to be implemented in each of the NIST CSF phases listed in Figure 4.

The NIST CSF phases are as follows:

- **Identify** threats and vulnerabilities;
- **Protect** critical infrastructure and minimize risk;
- **Detect** changes and anomalies;
- **Respond** to cyber incidents;
- **Recover** the previous state.

The NIS Directive requires each EU country to implement regulations for critical infrastructure sectors, including measures to secure IT/OT networks and information systems. Railway operators are classified as “Operators of Essential Services” (OES). Each EU member state was required to implement and enforce these measures by 2018. In fact, awareness and overall security have improved significantly. However, uneven implementation across member states, including a lack of enforcement, has been criticized. As a result, NIS 2.0 proposes the following measures to address these shortcomings:

- A common risk management strategy with a minimum list of basic security infrastructure to be applied;
- Common incident reporting procedures and timelines;
- Information security information sharing obligations;
- Cybersecurity risk assessment and mandatory training for management practices;
- Mandatory regulatory oversight and enforcement, including audits and random inspections;
- Penalties of up to one million euros, or 2% of the operator’s total annual worldwide profits.

### 4.3. Risk Management Mechanisms and Approaches

Passenger and freight trains are becoming more mobile as rail networks and traffic volumes expand, requiring increased protection. The rail system and rolling stock are perfectly safe, but accidents do occur, requiring the highest level of risk management expertise [27]. As NIS 2.0 fines approach GDPR [28] charges, cybersecurity of OT systems will become a major topic in future committee meetings. The question is, how much investment is required to effectively manage all these risks? The required investment is determined by the desired level of security and practices, as well as the organization’s risk tolerance. Traditional IT organizations typically spend 3–6% of their annual IT budget on cybersecurity risks, which include spending on security hardware and software, technical expertise and salaries, workflow development and maintenance, and so on. In the United States, the first cybersecurity regulations for rail transit will not be implemented until the end of 2021. Two cybersecurity mandates will be imposed by the federal government on “high-risk” rail systems and rail transit systems. The new security measures will require critical passenger and freight railroads to:

- Review cybersecurity risks to the federal government within 24 h;
- Assign a Cybersecurity Contact Person who is available 24 h a day, seven days a week;
- Create an incident response plan;
- Conduct a vulnerability analysis.

To achieve the appropriate level of sophistication for OT and IT risk management needs, they are complemented or coupled with other strategies. Their strategies must provide an acceptable level of compliance with local cybersecurity standards, and are linked to the organization’s broader enterprise risk management strategy. National rules under the NIS Directive have not been fully synchronized, which creates additional compliance issues for RUs and IMs operating in different EU Member States. Support from the railway sector is needed for all EU RUs and IMs to comply with the cybersecurity standards set by their national competent authorities. RUs and IMs depend on their suppliers to implement cybersecurity standards and to conduct more accurate vulnerability and threat assessments. Therefore, as the rail market transitions from a traditional, mechanical, and heavy industry to a more connected environment, it is necessary to rely on new reference documents and standards. In fact, there are multiple and diverse methodologies in use across the rail industry, but each may have a different scope and level of analysis. Although there are some regulations from other countries and sectors, such as ISO 2700X, IEC 62443, or the NIST (National Institute of Standards and Technology) cybersecurity framework, the requirements of the NIS Directive functions at the national level. The frameworks mentioned for operational technology systems were [4,29,30], or [31]. These guidelines and methodologies are often used in a complementary manner to effectively address either OT or IT infrastructure. OT systems require specialized techniques and frameworks designed for industrial railway systems, while IT systems are typically examined using broader and more generic techniques (such as *ISO 2700x or NIS Directive*). For example, the *ISA/IEC 62443 standards* are among the most frequently referenced frameworks for identifying specific OT assets and risks, although many of the report’s authors indicated that they plan to use the just-published *CLC/TS5070* in the future. National and international authorities have recommended a wide range of different strategies and techniques for managing cyber risk. The following section examines a selection of successful approaches from across Europe and around the world:

- *Standards ISO 27001, 27002, and 27005* [32]: One of the most popular and frequently cited families of information security guidelines is ISO 2700x (Information Security Standards). A system for managing information security within an organization is called an information security management system, while ISO 27001 is the standard for doing just that. The standards listed in ISO 27001 and 27002 should be considered when developing a risk management strategy. Risk management is emphasized in ISO 27005, which is used as an important guideline to manage and mitigate risks to an acceptable level by being able to perform a cybersecurity service from the beginning of the design activities (review of cybersecurity management plans, asset evaluation, incident management, etc.) to the testing activities (evaluation of white/gray/black box tests and review of attack scenarios and their consequences). The ISO27K series can be used for the business part of the railway infrastructure, which consists mainly of IT systems, according to CLC/TS 50701.
- *ISA/IEC 62443 specifications* [4]: A framework for addressing and minimizing security vulnerabilities in industrial automation and control systems (IACS) is provided by the ISA/IEC 62443 series of standards. They discuss details of industrial cybersecurity that are both technological and process-related, and provide a risk management strategy specific to OT systems that could be applied to OT in the railway industry. In particular, ISA/IEC 62443-3-2, “Security Risk Assessment, System Partitioning and Security Levels”, defines a series of technical measures to assist organizations in assessing the risk of a specific IACS and in defining and implementing security countermeasures to reduce that risk to an acceptable level. The IACS security zones and conduits presented in ISA/IEC 62443-1-1, Concepts and Models, is used as a key concept. The standard provides a basis for describing security countermeasures by matching the intended level of security with the required security level capabilities specified in ISA/IEC 62443-3, System Security Requirements and Security Levels.
- *CLC/TS 50701* [29]: Technical Specification 50701 (CLC/TS 50701, 2021) has been published in response to this standard. ISA/IEC 62443 is used in the railway industry according to this European standard. It applies to communications, signaling, processing, rolling stock, and fixed installations. It provides recommendations for models and concepts that can be used to develop requirements and guidelines to ensure that the residual risk from security threats is identified, monitored, and managed to an appropriate level by the party responsible for the railway network. A list of OT specific security measures and a list of OT components for the railway industry can be developed using CLC/TS 50701.
- *Shift2Rail Risk Assessment Methods (projects X2Rail-1 and X2Rail-3)* [30]: A risk assessment based on IEC 62443-3-2 has been proposed by Shift2Rail (X2Rail-1, 2019; X2Rail-3, 2020). It proposes a standard rail structure that includes:
  - A rail-focused attacker landscape;
  - Based on ISO 27005, ENISA’s 2016 Threat Taxonomy, and BSI: Threats Catalog, which is a threat environment for railways;
  - Impact Matrix;
  - Method for estimating security level targets and high-level risk assessment based on STRIDE threat classification [33];
  - Method for extensive risk analysis.
  A risk analysis of the typical railway signaling system was conducted by Shift2Rail in accordance with the established methodology. This analysis was carried out in order to comply with the standards set forth by the International Electrotechnical Commission (IEC) 62443. The analysis identified the target security levels for the different zones and proposed a simplified risk assessment strategy for the system in question. The proposed strategy included the following workflow:
  1. An overview of the zone to be assessed;
  2. A breakdown of the assessment into six STRIDE threat domains;
  3. Impact and likelihood assessment;
  4. Calculation of risk;
  5. Risk and security level mapping;
  6. Essential requirements;
  7. Security level mapping of baseline requirements to the six STRIDE threat domain security levels.
- *CyRail recommendations on cybersecurity of rail signaling and communication systems* [31]: In September 2018, the EU-funded CyRail7 project published a guidance document (CyRail, 2018). This guide presents an analytical examination of the potential hazards to rail infrastructure, outlining techniques for the identification and notification of attacks, strategies for the mitigation of risks, and profiles for safeguarding control and signaling applications. These measures are designed to reinforce the security of new rail infrastructure through the implementation of robust, comprehensive protection measures. The assessment is based on the internationally recognized standard, IEC62443, and comprises five key phases:
  - Identify the system under consideration;
  - Perform a high-level cybersecurity risk assessment to identify the most serious threats, areas, and channels, and identifying vulnerabilities;
  - Conduct a comprehensive risk assessment in perform a comprehensive risk assessment in each area and conduct key steps (classify threats, identify vulnerabilities, assess impact and consequences, calculate uncontrolled likelihood, quantify unmitigated cybersecurity risk, determine target security level, identify and assess available countermeasures, reassess likelihood and impact, calculate residual risk, and document and report results);
  - Record the process.
  Using the IEC62443 standard, these guidelines can be used to perform risk analysis in the railway industry, especially for control and signaling applications.
- *UIC Guidelines for Cyber-Security in Railways* [34]: The UIC ARGUS WG decided in 2018 to create an applicable documentation to give explicit instructions to the “railway” (International union of railways (UIC), 2018). This instruction is intended to help the railway sector to reduce its vulnerability to cyberattacks and to continuously ensure the availability, confidentiality, and integrity of railway data and systems. The report focuses specifically, but not exclusively, on signaling and communications in the rail industry. Based on the ISO 27001 and 27002 standards, it provides railway-specific advice. In addition, it outlines standard risk management procedures such as establishing the security context, identifying assets (both critical and non-critical), assessing impact (supported by operational impact scenarios), identifying threats, selecting appropriate threat scenarios, measuring the risk level for each relevant threat scenario based on its likelihood and impact, considering risk management solutions, and selecting from a variety of additional controls.
- *EULYNX, RCA, and OCORA approach* [35]: EULYNX is a European initiative led by 13 IM to standardize the interfaces and components of signaling systems. The EULYNX Reference Design outlines the general architecture, overarching architectural ideas, and all the generic functions of the system to define the overall EULYNX system. Baseline Set 3 was completed in 2020. RCA is an acronym for Reference Control, Command, and Signaling (CCS) Architecture. It is a starting point for EULYNX members and the ERTMS Users Group (EUG) to identify a common architecture for future railway CCS, with the primary objective of increasing CCS performance/total cost of ownership (TCO). The OCORA, RCA, and EULYNX Cybersecurity Guidelines have been added to the RCA Guideline Set 0 Release 1. It describes a risk assessment process based on the IEC 62443 and CLC/TS 50701 security standards and shows how and where to apply it to trackside CCS. The process is as follows:
  - System Definition;
  - Initial risk-based zoning concept;
  - An explanation of the different types of attackers;
  - Assessing adversary strength and motivation;
  - Threat supplementation;
  - Classifying threats into basic requirements;
  - Define the initial security level for each threat;
  - Incorporating the baseline requirement value into the initial zone vector;
  - Use reduction factors to calculate the final security level;
  - Implementing IEC62443 measures.
  The design of the CCS trackside is the main focus of RCA. OCORA, a related program, focuses on the architecture of the CCS on-board side. The next generation of European Train Control System (ETCS) on-board systems will have the architecture and interfaces defined by a cooperative initiative of five European railway companies.

The recent classification of railroads as “critical infrastructure” has precipitated a surge in the implementation of uniform, rail-oriented cybersecurity solutions. New protocols and other regulatory initiatives are motivating the industry to further reinforce its cybersecurity, including the adoption of standards such as CENELEC TS 50701 [36] (heavily based on IEC 62443) and recent TSA guidelines in the United States. However, this standardization is complicated by the diversity of players in the rail industry—operators, integrators, component suppliers, and external solution providers at various points in the supply chain. It is encouraging to note that stakeholders in the rail industry are taking steps to address the growing concerns surrounding cybersecurity. This is being achieved via innovation and the development of new products and services. Nevertheless, as cybersecurity becomes a paramount concern across the rail industry, it is imperative not to underestimate the challenges that lie ahead. These include the lengthy product lifecycle of rail items, the growing intricacy of rail systems, and the intrinsic necessity for transformation. Despite these obstacles, the future of the rail industry remains promising.

- There is a growing trend among operators in the industry to embrace a more cybersecurity-oriented culture. This involves greater transparency across the entire ecosystem, including visibility of activities among multiple vendors, operators, integrators, component suppliers and more.
- Railroad manufacturers and operators are required to maintain the implementation of robust cybersecurity measures, which must align with industry standards and be tailored to the specific requirements of the rail infrastructure. These measures must be consistently applied to new and existing systems, both on and off the rail.

### 4.4. Real-World Examples of Framework Application: Challenges and Limitations

In order to enhance this subsection on risk management in relation to the NIST Cybersecurity Framework and its application in railway systems, we propose the following suggestions:

1. The inclusion of *real-world examples of framework application* facilitates a practical understanding of the manner in which the NIST framework is implemented within the railway industry. The following examples serve to illustrate this point:
  - *Case Study: Metrolinx* (*Canada*) illustrates the integration of the NIST Cybersecurity Framework into the cybersecurity strategy of a regional transportation authority. Metrolinx conducted comprehensive risk assessments, which identified significant vulnerabilities in its operational technology and IT systems. This enabled the implementation of targeted mitigation strategies, enhancing the authority’s ability to protect against potential cyber threats while ensuring compliance with provincial regulations.
  - *Case Study: Network Rail* (*UK*) presents the utilization of the NIST framework by an organization operating the railway infrastructure in Great Britain to enhance its cybersecurity posture. It has undertaken risk assessments that align with the framework’s core functions: identify, protect, detect, respond, and recover. This has enabled Network Rail to enhance its incident response capabilities and establish a more robust defense against cyber incidents, particularly in light of increasing threats.
2. The examination of the *challenges and limitations* associated with implementing risk management frameworks. In order to provide a comprehensive analysis, it is necessary to consider a number of factors, including:
  - *Complexity of Integration*: The implementation of the NIST framework in railroad systems can be a challenging process, due to the necessity of integrating the framework with existing legacy systems. It is common for railway operators to use technologies that are no longer aligned with current cybersecurity standards.
  - *Resource Constraints*: The capacity for smaller rail operators to adopt comprehensive cybersecurity frameworks may be constrained by a combination of financial and human resource limitations. This could result in a lack of consistency in implementation across the industry.
  - *Evolving Threat Landscape*: The dynamic nature of cyber threats necessitates a continuous process of framework updating and refinement, which can be a costly undertaking for organizations.
3. A *comparative table of the various frameworks* that presents a comparison of significant elements and distinctions among an assortment of cybersecurity frameworks. The following layout is proposed in Table 9.

The integration of these components serves not only to reinforce this aspect of risk management, but also to furnish practical insights into the implementation of the NIST Cybersecurity Framework within railway systems.

### 4.5. Methodology Enhancement: A Proposal

**Figure 5:**
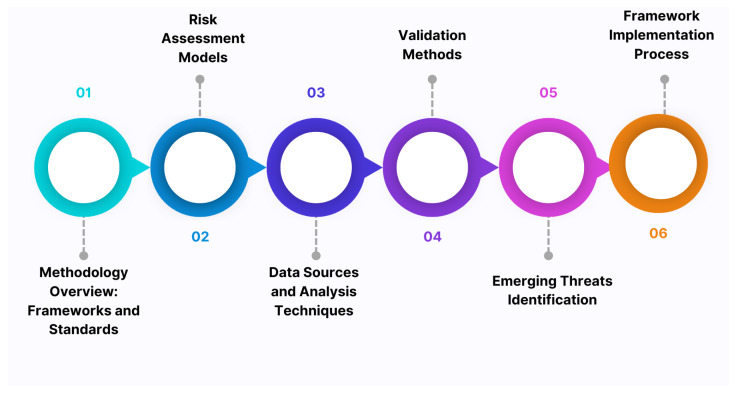
Methodology overview of the proposal.

In this paper, we will outline a methodology that has been developed for use in enhancing the existing/future approach to cybersecurity challenges in railway systems, as indicated in Figure 5. This approach incorporates structured frameworks, quantitative risk models and data validation techniques in order to provide a robust methodology for addressing these challenges.

The present paper addresses these points in considerable detail. For further information regarding Figure 5, please refer to the following:

1. *Frameworks and Standards:* The study incorporates internationally recognized cybersecurity frameworks, like IEC 62443 that provides a set of guidelines for the security of industrial control systems. Furthermore, the NIST Cybersecurity Framework provides a structured methodology for the identification, protection, detection, response to, and recovery from cyber threats. The integration process is illustrated as follows:
  (a) Identify Vulnerabilities: Conduct an evaluation of the system’s weaknesses in accordance with the criteria set forth in IEC 62443;
  (b) Evaluate potential threats in accordance with NIST standards, mapping them to the identified vulnerabilities;
  (c) Implement a layered security framework, comprising solutions such as network segmentation, firewalls, and AI-driven monitoring, in order to safeguard railway systems.
2. *Risk Assessment Models:* The quantification of risks is conducted through the utilization of the Common Vulnerability Scoring System (CVSS), which assesses both the probability and consequence of threats. Table 7 and Section 4.1 provide an illustrative example of the quantification and prioritization of risks.
3. *Data Sources and Analysis Techniques:* The following data sources may be generated for railroads based on:
  - Historical cyberattacks are subjected to analysis with the objective of identifying patterns and vulnerabilities;
  - Simulations: The generation of synthetic data allows for the testing of real-world scenarios;
  - AI-driven analysis: The identification of anomalies specific to railway operations.
4. *Validation Methods:* The efficacy of the proposed multi-layered framework must be substantiated through simulations and historical benchmarking to illustrate the mitigation success rates for various attack scenarios.
5. *Emerging Threats Identification:* The study employs a methodology that models threats such as APTs and IoT device vulnerabilities, with the objective of generating a heatmap of system vulnerabilities.
6. *Framework Implementation Process:* The proposed framework is designed in a way that allows for practical application and adaptation through a step-by-step methodology. The following steps must be undertaken during the implementation phase:
  (a) *System Audit*: Conduct an initial system audit in order to identify any potential vulnerabilities;
  (b) *Risk Prioritization*: The risk assessment matrix should be employed for this purpose;
  (c) *Foundational Security*: Implement foundational security measures, including the installation of firewalls and the implementation of patch management;
  (d) *Advanced Techniques Integration*: Integrate advanced techniques, such as the deployment of AI-driven detection tools and layered defenses.

By analyzing genuine case studies, the study identifies and addresses the challenges encountered, and provides an assessment of the strengths and weaknesses of various frameworks. This enhances both the clarity and value of this section. The objective of this paper is to provide a comprehensive overview of the manner in which these frameworks operate within the railway sector, and to highlight the importance of a robust cybersecurity posture in protecting critical infrastructure.

## 5. Emerging Threats and Future Security Trends

**Figure 6:**
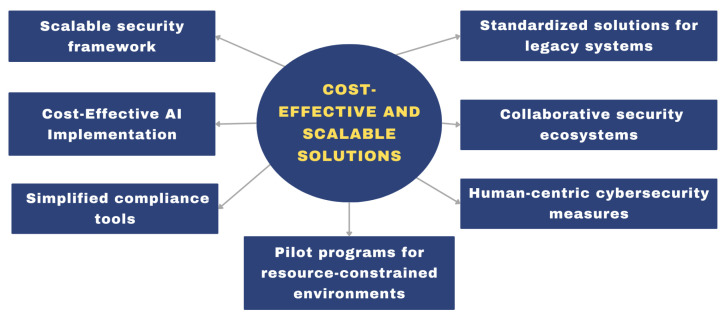
Cost-effective and scalable solutions.

The insights presented in the article indicate a need for future research to develop cost-effective and scalable cybersecurity solutions with adaptability to the diverse operational environments of railway operators, as shown in Figure 6. A key objective in this regard is ensuring the availability of accessible cybersecurity measures for railway operators, regardless of their size or resource availability. The goal is to achieve a balance between robust protection and practical deployment, fostering resilience across the global railway sector.

To contribute significantly to the existing body of knowledge in the field, the following areas represent potential avenues for future research:

- The use of *artificial intelligence* (*AI*) *and machine learning* for threat detection in railways. Recent advancements in these fields are transforming the way in which threats are identified in railway networks. These technologies facilitate real-time monitoring of rail systems, enabling the detection of anomalies in traffic and control signals to proactively address cyberattacks. For example, a study by Zhang et al. (2023) [37] demonstrated how AI models could be used to detect abnormalities in railway signaling systems and network traffic, thus minimizing potential operational disruptions due to cyberattacks. We can also recommend *Federated learning*, which enables the training of models on distributed data sources without the disclosure of the original data, thereby ensuring enhanced data protection and security. This approach could prove advantageous in contexts such as railway systems, where the analysis of data from diverse sources is necessary while preserving data confidentiality.
- The implementation of *Zero-Trust Architecture* is becoming increasingly prevalent within the railway industry [38]. This cybersecurity model guarantees that each and every connection, whether within or outside the network, is subjected to authentication and authorization procedures prior to granting access. As railway infrastructure becomes increasingly digitized, the implementation of Zero-Trust models is crucial for the prevention of unauthorized access to control systems. In 2022, several European railway operators initiated the integration of Zero-Trust architectures with the objective of safeguarding control centers and railway communication networks. It would be beneficial to examine the adoption of Zero-Trust principles by railway operators and the ways in which they are utilized to deter unauthorized access to sensitive systems, such as signaling or maintenance systems [39].
- *5G Network Integration* and Security in Railways with the goal of improving real-time data transmission. The implementation of 5G enables faster communication between trains, control centers, and maintenance systems. Nevertheless, the advent of 5G also presents novel security issues, including the potential for exploitation of real-time communication links through cyberattacks [40]. For instance, a report published in 2023 by the European Union Agency for Cybersecurity (ENISA) identified potential risks related to the integration of 5G networks into railway systems. These risks, which could have severe consequences for train operations, include the possibility of Denial of Service attacks. It would be valuable to consider how the adoption of 5G technology might affect railway security, and to explore how railway operators are addressing these concerns through the implementation of secure access control protocols, data encryption, and network segmentation.
- The implementation of *hybrid cloud architectures* enables railway operators to achieve a balance between the necessity for scalability and the imperative for information security. It is imperative that research be undertaken to investigate the implications of transferring sensitive data to cloud environments. This should encompass the areas of encryption, access control, and adherence to the relevant regulatory standards.
- The advent of *quantum computing* has introduced a significant long-term challenge to the efficacy of conventional encryption techniques employed in railway communication and control systems. In response, the railway industry is actively investigating the potential of quantum-resistant encryption algorithms to safeguard sensitive data against future-proof threats. It is recommended that the discussion address the growing importance of quantum-resistant encryption as a crucial frontier in railway cybersecurity, with a view to protecting communication channels from the potential impact of quantum-based decryption attacks in the coming decades.
- *Advanced incident response and recovery plans* are necessary to ensure the minimal disruption to railway operations. Recent incidents have demonstrated that many railway operators lack comprehensive incident response plans. These must include predefined steps for the isolation and containment of incidents affecting operational and customer-facing systems. Furthermore, the implementation of cyber resilience testing and disaster recovery drills is recommended. This will facilitate the rapid recovery of railway systems following a cyberattack, thus minimizing downtime and protecting the public [27].
- *Blockchain technology* can be implemented to ensure the integrity of data, particularly data derived from sensors. In critical environments such as those found in the rail industry, it is of paramount importance to guarantee the veracity of data and to provide a record which cannot be altered without being noticed. The use of blockchain can provide a decentralized ledger which can be used to track all data communication between sensors and control systems, thus ensuring that data are not compromised over time.
- *Advanced Authentication Mechanisms*: It is recommended that behavioral biometrics or context-aware authentication methods that combine user behavior with other factors (e.g., geolocation) be investigated in order to ensure a more secure and seamless authentication process. This may be achieved by exploring next-generation authentication mechanisms in the context of the Internet of Things (IoT) and smart transportation systems.
- *Security-by-Design Frameworks*: It is imperative to emphasize the significance of security-by-design methodologies [41], wherein cybersecurity is seamlessly integrated into the design and development of railway infrastructure systems. This necessitates adherence to the Secure Software Development Life Cycle (SDLC) and the deployment of risk management frameworks from the nascent stages of project design.

**Figure 7:**
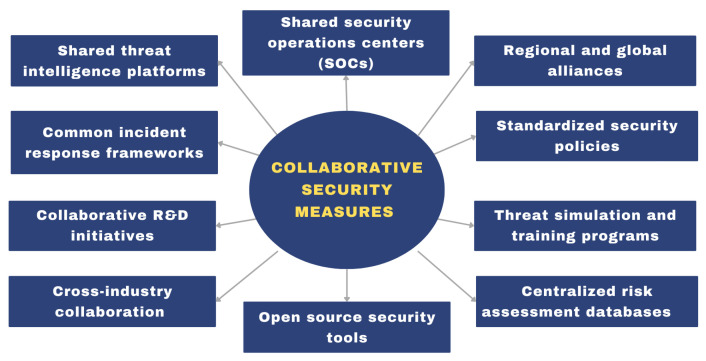
Collaborative security measures.

In railway infrastructures context, it is strongly recommended to consider:

- *Collaborative security measures:* These measures are critical for monitoring and responding to threats in real time across rail networks. These measures leverage shared resources, intelligence, and expertise to effectively and efficiently address threats and ensure a unified approach to cybersecurity. Figure 7 shows some recent key recommendations for improving rail cybersecurity collaboration.
  By combining resources and expertise, railway networks can monitor threats in real time, respond quickly to incidents, and ensure a robust security position across connected systems. These activities are essential to ensure the integrity and safety of rail traffic worldwide. By fostering a collective defense against cyber threats, collaborative security measures strengthen the resilience of individual operators, which can start with:
  1. *Implementing shared tools and platforms:* Start with the simplest measures, such as threat intelligence platforms, that provide a high level of value at a relatively low cost of implementation;
  2. *Encouraging regional collaboration:* Regional SOCs and alliances can help streamline the coordination between operators located in the same geographic area;
  3. *Investing in standardization and R&D:* Long-term initiatives, such as standardized policies and joint R&D initiatives, provide alignment and a sustained innovative impact.
- *Collaborate with Industry and Governmental Bodies*: The issue of cybersecurity for critical infrastructures like railways cannot be solved by a single organization; rather, it requires a collaborative approach that draws on the resources and expertise of government agencies, industry bodies, and international organizations. By pooling knowledge and sharing intelligence, organizations can enhance their collective resilience and stay informed about emerging threats, which is particularly important in the context of rapidly evolving cyber threats. The value of collaboration within an organizational context reveals that it enables organizations to harness collectively held knowledge and capabilities. This ensures that even the most sophisticated threats can be addressed through joint efforts, thereby fostering a more secure ecosystem for critical infrastructure systems globally.
- A comprehensive cybersecurity program for railway personnel must take into account the increased digitization of railway systems and the continued vulnerability of the *human factor*. It is imperative that all personnel, including operators, engineers, and administrative staff, undergo continuous and adaptive cybersecurity training in order to prevent social engineering attacks and ensure compliance with cybersecurity protocols. It would be beneficial to emphasize the importance of regular phishing simulations, security awareness workshops, and role-based training for all staff involved in railway operations, with the aim of minimizing the risk of insider threats or accidental security breaches.

The incorporation of these solutions adds depth and credibility to the article, situating our research within the existing literature and suggesting novel approaches to addressing pivotal challenges. This broader perspective will not only elucidate existing challenges but also inform future research directions in this critical field.

## 6. Conclusions and Perspectives

The present study offers a comprehensive overview of the contemporary cybersecurity challenges confronting railway systems, with a specific emphasis on the susceptibility of sensor and control system networks. Through an examination of extant cybersecurity frameworks, including those delineated by NIST and the IEC 62443 standard, and a proposal of pivotal strategies, such as network segmentation, encryption, and AI-driven threat detection, this document elucidates pivotal domains wherein enhancements must be implemented in order to effectively mitigate the evolving cyber risks to which railway systems are exposed. Nevertheless, the practical application of these solutions remains a significant challenge due to the limitations of existing infrastructure, the constraints of available resources, and the technical obstacles that must be surmounted. Future research should concentrate its efforts on developing solutions that can be readily implemented by a diverse range of railway operators, while also being cost-effective and scalable. Moreover, as technologies such as AI and IoT continue to evolve, it becomes increasingly vital to conduct ongoing research to understand how emerging threats may leverage these advancements and to develop resilient and adaptive defense mechanisms. In addition to this, future research should investigate the potential benefits of collaborative security initiatives that enable real-time threat monitoring and response across railway networks, with the aim of ensuring the continued safety and efficiency of global railway systems. 

## Figures and Tables

**Table 1:** Timeline of some cybersecurity incidents in the railway sector.

Date	Description
August 2003	Thousands of kilometers of railroad signals were affected when a computer virus shut down the CSX Railway headquarters in Florida [7].
January 2008	After hacking into the train system in Lodz, Poland, a teenager derailed four tram cars, injuring twelve people [8].
December 2012	Train signals were interfered with for two days by a cyberattack on the computers of a Northwestern US rail firm [9].
March 2015	In the course of six weeks, the HoneyTrain Project counted 2,745,267 login tries with four successful unauthorized accesses to a human–machine interface (HMI) of a virtual train control system [10].
February 2016	A well-known Ukrainian rail company’s computers were attacked with the malware BlackEnergy and KillDisk [11].
July 2016	According to a study, at least four big cyberattacks, including intrusions into the actual train system, had a negative impact on the UK Network Rail during the course of a year. These attacks appeared to be exploratory [12].
November 2016	The San Francisco light rail transit system’s (SF Muni) ticket machines were out for a day due to a ransomware attack; however, the service, security systems, and the personal information of passengers, were unaffected [13]
May 2017	A ransomware attack on the data systems of Deutsche Bahn occurred [12]. Russian [14] and Chinese [15] national railway networks were also affected by the same computer malware.
October 2017	A DDoS attack on the IT systems that track railroad activity was directed at Sweden’s transportation authority. The following day, Vasttrafik, a provider of public transportation, was subject to two DDoS attacks [12].
May 2018	A DDoS attack on the Danish operator DSB made ticket purchases impossible. The DSB staff’s internal mail and phone systems were also compromised [16].
October 2020	The Montreal transportation company (STM) was the target of a ransomware attack that compromised 624 servers that were crucial to operations. STM was also impacted by the outage for more than a week [17].
December 2020	OmniTRAX was the target of a ransomware assault. It was the first instance of the so-known double-exhortation ransomware assault against a US freight rail company to be made public [18].
December 2020	TransLink had to shut down various IT services, including a portion of its payment systems, as a result of the Egregor ransomware attack [19]. Neither transit safety system was damaged, although bus GPS capabilities were impacted by IT issues [20], and personal banking and social insurance information may have been exposed [21].
July 2021	Iran’s railroad system was the target of a cyberattack that destabilized the entire nation [22].
October 2021	The Toronto Transit Commission (TTC) was the target of a ransomware attack, and as a result, it was unable to access systems for online booking and to communicate with vehicle operators [23]. The TTC then disclosed that (former) employees’ personal information may have been stolen [24].

**Table 2:** An expanded view of recent case studies in railway cybersecurity.

Incident	Type of Attack	Downtime (Hours)	Financial Impact (EUR)	Mitigation Applied	Effectiveness (%)
Deutsche Bahn (2017)	Ransomware	24	10 million	Network segmentation, employee training	85
European Rail (2021)	Phishing	16	6 million	MFA, anti-phishing software	90
Japanese Railways (2022)	IoT Device Hijack	48	12 million	Secure IoT gateways, AI threat detection	80

**Table 3:** A classification of cybersecurity issues and problems in the railways.

**Technology** (Technological requirements—related issues)	Cybersecurity	Handling, operation, and maintenance of secure systems
	Cost trade-off	Decisions made during the design phase may have long-term effects and be challenging to predict
	Data Volume	Challenges with data collection, transmission, storing, and analysis due to the general amount of traffic
	Data Silos	Reduces decision making process effectiveness due to data availability restrictions
	Backward compatibility	To sustain system accessibility and data exchange, backward compatibility is necessary
	Data formats	Lock-in with specific data formats and incompatible storage systems and exchange formats
	Technical constraints	Partners’ incompatibility with one another’s domain needs, data processing methodologies, and system technologies
	Technical risk	Risk due to a lack of understanding of system complexity and the limitations of technology
	Awareness	Knowledge of technological concerns and problems that have been solved, and available technologies
**Governance** (Regarding elements of the system’s general governance)	Regulation	Outlining the procedures for managing data
	Democratization	Provides equitable rights, opportunities, and guidelines for data access
	Legislative aspects	Legal agreements outlining data access and the application of such agreements
	Safety	Data gathering, processing, analysis, exchange, and other aspects of data safety
	Confidentiality	Organizational responsibility to protect data confidentiality
	Privacy	Individuals have a right to keep their personal information private
	Fairness	Information, risk, and rewards are distributed fairly among all stakeholders
	Transparency	Source verification and transparency in data gathering, use, and sharing
	Authentication and authorization	Transparent, standardized authentication and authorization procedures for physical and cyber systems
	Ownership	Establishing, leasing, and transferring ownership with auditable records
	Integrity	Authenticated source, secure data and metadata storage and transfer, auditable record keeping, and ownership
	Proprietary systems	Organizational requirements are indispensable, and incompatible with stakeholders
	Organizational requirements	Requirements to further the goals and interests of the organization
	Standards	Necessary for interoperability, scale economies, and accelerating research
	Upgradability	Mechanism to perform unsupervised software upgrading that is secure, durable, and robust
	Awareness	Knowing one’s objectives, plans, and activities both short- and long-term
**Business** (Regarding aspects of company operations)	Relative advantage	Benefits of new technology over existing techniques, which may not just be in the technical field
	Compatibility	Not merely the digitalization of existing processes, but also compatibility with and improvements to existing processes
	Complexity	Complicated in regard to resource availability, technology adaptation, and transition costs
	Trialability	Analyze and implement solutions to well-known issues to improve usability
	Observability	Benefits that are evident and quantifiable results from implementing new technology
	Awareness	Understanding the fundamentals of core business practices

**Table 4:** Anticipated incidence and impact.

Year	Estimated Incident Frequency	Average Financial Impact per Incident (EUR)	Total Annual Impact (EUR)
2024	50	500,000	25 million
2025	65	600,000	39 million
2026	80	750,000	60 million

**Table 5:** Potential challenges and limitations of strategy implementation.

Asset	Challenge/Limitation	Solution/Mitigation
Legacy Systems	A considerable number of railway systems are currently in operation that were constructed with legacy infrastructure. Such infrastructure may not be capable of supporting the latest encryption standards or network segmentation practices. Consequently, the upgrading of these systems may prove to be both costly and time-consuming.	It is recommended that a phased approach be adopted, with critical systems being upgraded first. Gateway solutions that facilitate secure communication between legacy and modern systems through protocol translation and firewalling should also be introduced.
Cost and Resource Constraints	The implementation of sophisticated security procedures, such as AI-driven threat detection and blockchain technology for data integrity, can be financially onerous, particularly for smaller organizations.	It is recommended that organizations begin by implementing low-cost, high-impact solutions, for example, regular staff training on the subject of phishing attacks and multifactor authentication. As budget and resources permit, the implementation of further advanced solutions should be considered.
Real-Time Data Encryption	While encryption provides protection for data in transit, it may introduce latency issues in real-time systems where rapid decision making is essential, such as train control.	It is recommended that lightweight encryption algorithms or hardware-accelerated encryption be used in order to minimize the impact on performance.
Integration with Existing Frameworks	The integration of novel security measures with existing frameworks, such as the International Electrotechnical Commission (IEC) 62443 or the National Institute of Standards and Technology (NIST) standards, can prove challenging due to the discrepancies in protocols and technology stacks.	It is essential to collaborate with vendors and consultants in order to guarantee that the security solutions in question are capable of functioning in conjunction with existing frameworks and systems. Prior to the complete deployment of the aforementioned solutions, it is vital to conduct comprehensive interoperability testing in order to identify and resolve any potential integration issues.
AI-Driven Threat Detection	It is challenging to effectively identify anomalies using AI and machine learning models in railway systems, where large-scale cyberattacks are rare, due to the necessity for large datasets.	In order to achieve a comprehensive security strategy, it is recommended to combine artificial intelligence (AI) with traditional security mechanisms. These may include firewalls and intrusion prevention systems (IPS), which can be integrated to create a multi-layered defense.
Scalability	It may be challenging to implement solutions such as AI-driven monitoring and blockchain across national or multinational railway networks, due to their inherent complexities. The implementation of advanced solutions often necessitates the regular application of updates, patches and monitoring, which may prove onerous for existing IT teams.	It would be beneficial to consider outsourcing certain aspects of cybersecurity to a third-party managed security service provider (MSSP), given their capacity for undertaking comprehensive monitoring and management of complex IT infrastructures.
User Resistance and Human Factors	The implementation of novel security protocols, such as multifactor authentication (MFA), may encounter resistance from employees who perceive it as an unduly onerous procedure.	It is recommended that training and awareness campaigns be conducted in order to elucidate the significance of cybersecurity measures for the protection of the railway system’s integrity. Additionally, the utilization of user-friendly tools and the simplification of security processes are advised in order to reduce friction and enhance overall efficiency.

**Table 6:** Return on investment for cybersecurity measures.

Challenge/Measure	Implementation Cost (EUR)	Estimated Savings (EUR)	ROI (%)	Notes
Legacy Systems	100,000	300,000	200%	Reduce vulnerability to legacy systems with secure gateways and incremental upgrades.
Cost and Resource Constraints	50,000	200,000	300%	Leverage lightweight security protocols designed for constrained environments.
Real-Time Data Encryption	150,000	600,000	300%	Encrypt sensitive data with quantum-resistant encryption.
Integration with Existing Frameworks	75,000	250,000	233%	Adaptation of new security measures to existing IT and OT frameworks such as the IEC 62443 standard.
AI-Driven Threat Detection	200,000	1,000,000	400%	Reduce response times and prevent breaches with AI-powered anomaly detection for security and compliance.
Scalability	120,000	400,000	233%	Designing modular solutions that can be expanded to meet the needs of the operator without major overhauls.
User Resistance and Human Factors	30,000	150,000	400%	Reduce insider threats and improve adoption by implementing training and awareness programs.

**Table 7:** An example for quantifying and prioritizing risks.

Vulnerability	Threat	Impact (1–5)	Likelihood (1–5)	Risk Score (Likelihood x Impact)	Priority
Unpatched Legacy Systems	Exploitation of Known Vulnerabilities, Ransomware, Data Breaches	5	4	20	High
Weak Authentication	Credential Stuffing, Phishing, Brute Force Attacks	4	5	20	High
Insider Threats	Data Theft, Sabotage, Privilege Escalation, Social Engineering	3	3	9	Medium
Lack of Security Awareness	Social Engineering, Phishing, Negligence	3	2	6	Medium
Unmonitored IoT Devices	IoT Breaches, Unauthorized Access	4	3	12	Medium
Inadequate Network Segmentation	Lateral Movement, Data Exfiltration, Ransomware	5	2	10	Medium
Outdated Security Protocols	Protocol Downgrade Attacks, MITM (Man-in-the-Middle)	4	2	8	Low
Limited Incident Response Capabilities	Delay in Breach Detection, Unresolved Attacks	3	1	3	Low

**Table 8:** Various cybersecurity frameworks.

**(ISC)2 Certified Information**	**ISO 27001/27002v2022**	**NIST SP 800-53 Revision 5**	**CIS Critical Security Controls v8**
**Systems Security Professional**	93 controls in 4 domains	322 controls in 20 families	18 security controls
**(CISSP)**—8 domains	Last update: October 2022	Updated December 2020	Last update: May 2021
Last update on May 2021			
1. Security and Risk	1. Organizational controls	1. Access control	1. Inventory and Control of Enterprise
Management	(37 controls)	2. Awareness and training	Assets
2. Asset Security	2. Technological controls	3. Audit and accountability	2. Inventory and Control of Software Assets
3. Security Architecture	(34 controls)	4. Assessment, Authorization,	3. Data Protection
and Engineering	3. Physical controls (14	and Monitoring	4. Secure Configuration of Enterprise Assets
4. Communication and	controls)	5. Configuration management	and Software
Network Security	4. People controls (8	6. Contingency planning	5. Account Management
5. Identity and Access	controls)	7. Identification and authentication	6. Access Control Management
Management		8. Incident response	7. Continuous Vulnerability Management
6. Security Assessment		9. Maintenance	8. Audit Log Management
and Testing		10. Media protection	9. Email and Web Browser Protections
7. Security Operations		11. Physical and environmental protection	10. Malware Defenses
8. Software Development		12. Planning	11. Data Recovery
Security		13. Program Management	12. Network Infrastructure Management
		14. Personnel security, Processing and	13. Network Monitoring and Defense
		Transparency	14. Security Awareness and Skills Training
		15. Personally Identifiable Information	15. Service Provider Management
		16. Risk assessment	16. Application Software Security
		17. System and service acquisition	17. Incident Response Management
		18. System and communications protection	18. Penetration Testing
		19. System and information integrity	
		20. Supply Chain Risk Management	

**Table 9:** A comparative table of the various frameworks.

Framework	Key Features	Focus Area	Limitations
NIST Cybersecurity Framework	The system is designed to facilitate comprehensive risk management, offering flexibility and customization. Its core functions, which are identified, protected, detected, responded to, and recovered from, are integrated into a unified framework.	Critical infrastructure, including railways.	The complexity of implementation in legacy systems represents a significant challenge.
ISO/IEC 27001	The information security management system (ISMS), risk assessment, and subsequent treatment are the three key elements of this process.	The safeguarding of information across a diverse range of sectors.	It is possible that the procedure may entail the necessity for comprehensive documentation and auditing procedures.
CIS Controls	The most effective practices and actions that can be undertaken to ensure cybersecurity.	A general overview of cybersecurity management.	Less comprehensive in relation to specific industries.
IEC 62443	Cybersecurity for industrial automation and control systems is concerned with the management of risks and the fulfillment of security level requirements.	Industrial control systems, including railway.	Focuses predominantly on industrial systems, and consequently, may lack sufficient attention compared to other organizational aspects.

## Data Availability

As this is a review paper, no new data were generated or analyzed. All data referenced in this review are publicly available through the sources cited in the manuscript.

## Abbreviations

AI	Artificial Intelligence
APT	Advanced Persistent Threats
CAN	Controller Area Network
CISSP	Certified Information Systems Security Professional
CPS	Cyber–Physical Systems
CVSS	Common Vulnerability Score System
DoS	Denial of Service
ETCS	European Train Control System
EUG	ERTMS Users Group
HMI	Human–Machine Interface
IACS	Industrial Automation and Control Systems
IDS	Intrusion Detection Systems
IEC	International Electrotechnical Commission
UIC	International Union of Railways
ISMS	Information Security Management System
ISP	Internet Service Provider
IT	Information Technology
IPS	Intrusion Prevention Systems
ISO	Information Security Standards
MFA	Multi-Factor Authentication
MQTT	Message Queuing Telemetry Transport
MSSP	Managed Security Service Provider
MVB	Multifunctional Vehicle Bus
NIST	National Institute of Standards and Technology
OES	Operators of Essential Services
OT	Operational Technology
RBAC	Role-Based Access Control
ROI	Return On Investment
SIEM	Security Information and Event Management
TPM	Trusted Platform Module
VLAN	Virtual Local Area Networks
